# Recent Advances in the Isolation, Synthesis and Biological Activity of Marine Guanidine Alkaloids

**DOI:** 10.3390/md15100324

**Published:** 2017-10-24

**Authors:** Jin Liu, Xu-Wen Li, Yue-Wei Guo

**Affiliations:** 1 State Key Laboratory of Drug Research, Shanghai Institute of Materia Medica, Chinese Academy of Sciences, 555 Zu Chong Zhi Road, Zhangjiang Hi-Tech Park, Shanghai 201203, China; ljin080@mail.ustc.edu.cn; 2 Nano Science and Technology Institute, University of Science and Technology of China, 166 Ren Ai Road, Suzhou 215123, China

**Keywords:** marine guanidine alkaloids, sponge, mollusk, ascidian, total synthesis, biological activity

## Abstract

Marine organisms are prolific resources of guanidine-containing natural products with intriguing structures and promising biological activities. These molecules have therefore attracted the attention of chemists and biologists for their further studies towards potential drug leads. This review focused on the guanidine alkaloids derived from marine sources and discussed the recent progress on their isolation, synthesis and biological activities, covering the literature from the year 2010 to the present.

## 1. Introduction

The marine environment has been regarded as one of the most complex biospheres on earth due to its huge ranges of light (complete darkness to extensive brightness), temperature (−2 °C to over 300 °C), pressure (1 to more than 1000 atmospheres), nutrient conditions (sparse to rich), etc. Such circumstances enable the existence of numerous species to produce extremely diverse and complex secondary metabolites with a wide range of significant biological activities [1]. Over the past few decades, at least nine marine natural product (MNP)-derived drugs have been successfully developed, of which seven are nitrogenous compounds [2]. Therefore, although they have only been studied for over half a century, MNPs have already shown great potential in new drug discovery.

**Figure 1:**
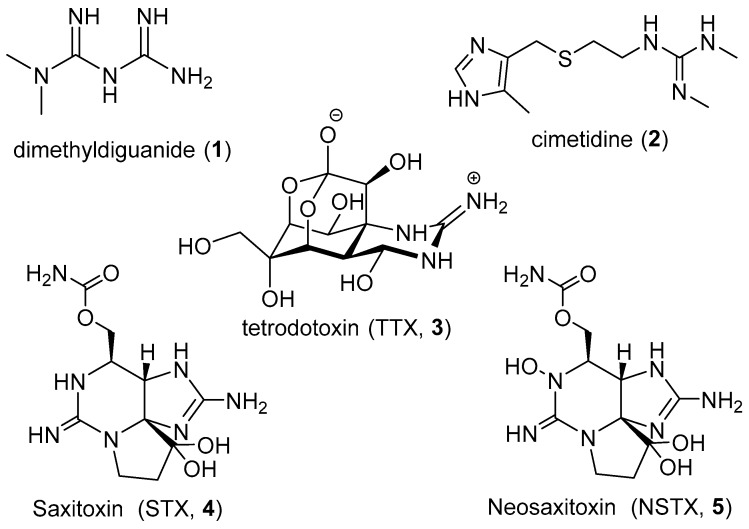
Structures of the represented guanidine compounds containing drugs or toxins.

Guanidine is considered to be one of the strongest organic bases (p*K*_a_ = 13.6) [3]. Such a basic property enables this molecule to bind to carboxylates, phosphates and metals. Its guanidinium cation can engage in the special interactions between ligand/receptor or enzyme/substrate [4]. Therefore, the guanidine group is of great interest in medicinal chemistry and has become a key motif in many clinical drugs such as the famous anti-diabetic drug dimethyldiguanide (**1**) and the peptic ulcer drug cimetidine (**2**) (Figure 1). Guanidine derivatives are widely distributed in nature and have attracted a lot of attention due to their chemical diversity and broad biological activities. Due to this fact, several groups have summarized the chemical and biological characteristics of natural guanidine products with different emphases and scopes. For instance, Chen and co-workers published a review paper in *Tetrahedron* in 2015, which mainly discussed the synthesis of cyclic guanidine-containing natural products [5]. Netz and Opatz published a general review of marine indole alkaloids from 2003 to 2015, in which some guanidine compounds were also included [6]. Berlinck and co-workers have been continuously reviewing the chemistry and biology of natural guanidine products in the journal *Nat. Prod. Rep.* since 1996, and the most recent one in 2016 [7] summarized some isolation and synthesis of marine guanidine compounds from 2012 to 2014, including guanidine peptides and alkaloids. In this review, peptides will not be discussed, while the recent (2010–2017) advances in guanidine alkaloids from marine sources will be specifically focused on, aiming to provide insight into the further discovery and preparation (e.g., synthesis) of interesting bioactive marine guanidine compounds for future medicinal applications.

Marine organisms are the richest sources of natural products that have guanidine functionality, which is probably due to their special living environments. Many well-known toxins with guanidine groups have been found in marine resources, such as tetrodotoxin (TTX, **3**) from tetraodontidae (puffer fishes) [8], saxitoxin (STX, **4**) from shellfish [9], and neosaxitoxin (NSTX, **5**) from algae (Figure 1) [10]. These toxic marine natural products have high medical research value. In recent years, the impressive diversity and complexity of cyclic and/or linear guanidine MNPs were observed as PKS (polyketide synthase) and/or NRPS (nonribosomal peptide synthetase) derivatives, terpene and/or peptide conjugates, etc. [7,11]. These metabolites were mainly derived from various beautiful marine sponges, mollusks, and ascidians in different locations. This review will summarize the recent progress on the isolation, synthesis and biological activities of marine guanidine alkaloids (MGAs) according to their different origins.

## 2. MGAs from Sponges

Although many reports of guanidine alkaloids from marine algae, mollusks, microorganisms, etc., the richest sources of such molecules are marine sponges. To the best of our knowledge, during the last 7 years, over 90% MGAs were discovered in sponges, such as those of the genera *Monanchora*, *Crambe*, *Clathira*, *Acanthella*, *Halichondria*, etc. Among these, *Monanchora* was the most popular genus to have been chemically investigated, with diverse and complex cyclic or linear MGAs being isolated and determined. Accordingly, this section will be described based on the MGAs from different sponge genera [7,11].

### 2.1. Monanchora

*Monanchora*, of the order Poecilosclerida and the family Crambeidae, is one of the richest sources of MGAs, especially complex polycyclic guanidines, such as crambescidins, ptilomycalins, fromiamycalins, celeromycalins, batzelladines, monanchomycalins, monanchocidins, normonanchocidins, neofolitispates, etc. Most of the recently reported MGAs were derived from this genus, with a wide range of biological activities such as cytotoxic, antimicrobial, antimalarial, and anti-HIV properties [12].

**Figure 2:**
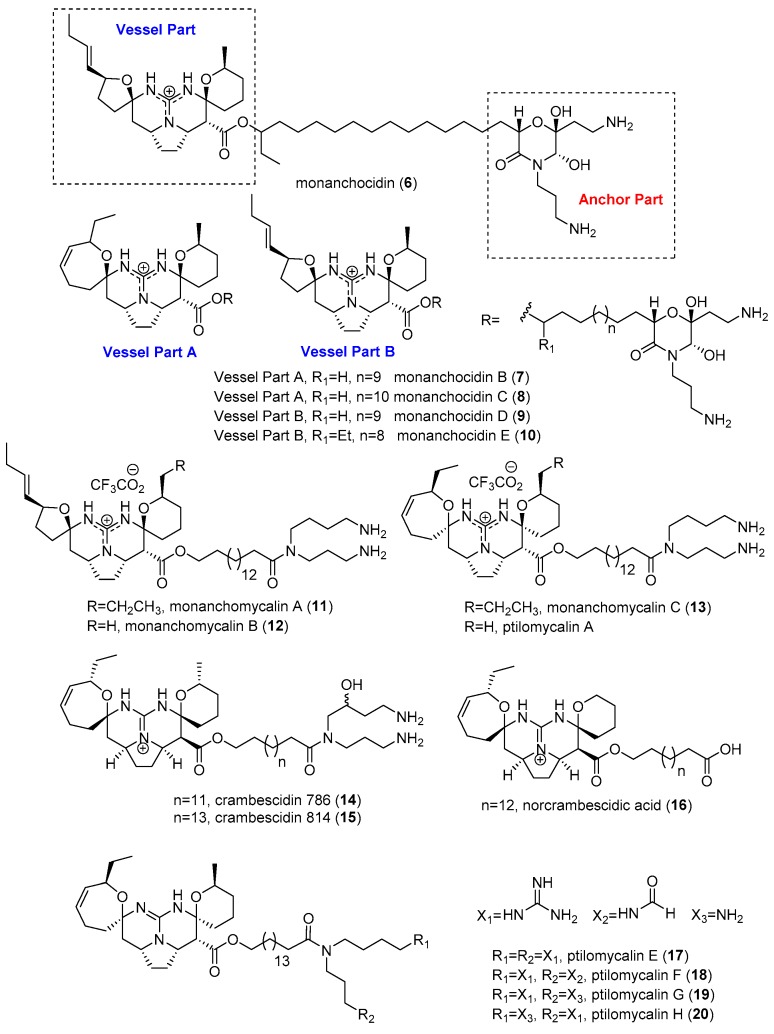
Structures of pentacyclic MGAs (**6**–**20**) from sponges of the genus *Monanchora*.

Pentacyclic MGAs are one of the most complex marine guanidine-containing natural products that have intriguing structures (Figure 2). Monanchocidin (**6**) is a novel pentacyclic guanidine derivative that was isolated by Makarieva and co-workers in 2010 from the sponge *M. pulchra,* collected near Urup Island at a depth of 66 m [13]. In fact, the novelty of this molecule is the presence of an unprecedented bis-spiro-pentacyclic guanidine ring system (including two contiguous 1-oxa-6-azaspiro[4,5]decane and 1-oxa-7-azaspiro[5,5]undecane spiro-ring systems), a long chain moiety derived from a polyketide precursor (ω-3)-hydroxy fatty acid, and a 2-aminoethyl- and 3-aminopropyl- substituted morpholine hemiketal ring. Thus, monanchocidin is believed to be a PKS-derived MGA. Biological evaluation revealed that **6** exhibited cytotoxicity against human leukemia THP-1, human cervix epithelioid carcinoma HeLa, and mouse epidermal JB6 Cl41 cell lines, with IC_50_ values of 5.1, 11.8, and 12.3 μM, respectively. This derivative also showed 66% apoptosis-inducing effect in THP-1 cells at a concentration of 3.0 μM. In the following year, the same author discovered a series of monanchocidin analogs from the same species collected in the same location, including **6** [14]. In order to avoid future confusion, they re-designated “monanchocidin” as “monanchocidin A”. The four new isolates were named monanchocidins B–E (**7**–**10**). Among the five compounds, **6**, **9**, and **10** shared the most similar structures, with the only difference being the length of the linked chain (14 for **6** and **9**, 13 for **10**) between the pentacyclic guanidine ring and the morpholine hemiketal ring or the loss of an ethyl group on C-23 position for **9**. Monanchocidins B and C (**7** and **8**) differed from **6** by the replacement of 1-oxa-6-azaspiro[4,5]decane in **6** with an 1-oxa-8-azaspiro[6,5]dodecane in **7** and **8** in the bis-spiro-pentacyclic ring system. In fact, the pentacyclic ring system of **7** and **10** was the crambescidin skeleton. All five MGAs **6**–**10** displayed strong cytotoxicity against human promyelocytic leukemia HL-60 cells with IC_50_ values of 540, 200, 110, 830, and 650 nM, respectively. Intriguingly, for the structures of this series of compounds, Kashman [15] pointed out that the bis-spiro-polycyclic alkaloids look like a vessel, and Makarieva referred to the morpholine hemiketal ring of compounds **6**–**10** as an anchor; thus, the long hydrocarbon chain could act as an anchorage cable to link both vessel and anchor.

In 2012, two new monanchocidin analogs, monanchomycalins A (**11**) and B (**12**) from the deep sea sponge *M. pulchra* were isolated by Makarieva’s group near Urup Island at a depth of approximately 150 m [16]. Their structures differed from that of monanchocidin A mainly by the variation of the “anchor” moiety. In fact, a spermidine residue acted as the anchor in these compounds instead of the morpholine hemiketal ring in monanchocidins. Monanchomycalins A (**11**) and B (**12**) exhibited stronger cytotoxicity than the previous analogs, with IC_50_ values of 120 and 140 nM, respectively, against HL-60 cells. Monanchomycalin C (**13**), an analog of the above two MGAs, was isolated by the same group from the same species in the sea near Kunashir Island in 2013 at a depth of 114 m [17]. It was found to be cytotoxic against human breast cancer MDA-MB-231 cells with an IC_50_ value of 8.2 μM. Most recently, in 2016, Ali and co-workers isolated three crambescidin derivatives, i.e., crambescidin 786 (**14**), crambescidin 814 (**15**), and norcrambescidic acid (**16**), from a French Polynesian *Monanchora* n. sp. sponge collected off the coast of Hiva Oa at a 20-m depth [18]. All three compounds exhibited significant cytotoxic activities against KB tumor cell lines (a subline of the ubiquitous KERATIN-forming tumor cell line HeLa), with IC_50_ values of 0.3, 0.005, and 0.5 μM, respectively. In 2017, Gauvin-Bialecki and co-workers discovered four ptilomycalin derivatives, i.e., ptilomycalins E–H (**17**–**20**), from the sponge *M. unguiculate* collected at 2–6 m in Mitsio Islands, Madagascar [19]. Structurally, all these compounds comprise the cyclic guanidine group as well as one or two acyclic guanidine groups as the “anchor” moiety. It is worth mentioning that isomers **19** and **20** were obtained as a 2:1 mixture, which could not be separated but could be structurally identified by the authors. Biologically, **17** and the mixture of **19**/**20** showed strong cytotoxicity against KB cell lines with IC_50_ values of 0.85 and 0.92 μM, respectively, while **18** displayed promising activity against *Plasmodium falciparum* with an IC_50_ value of 0.23 μM.

**Figure 3:**
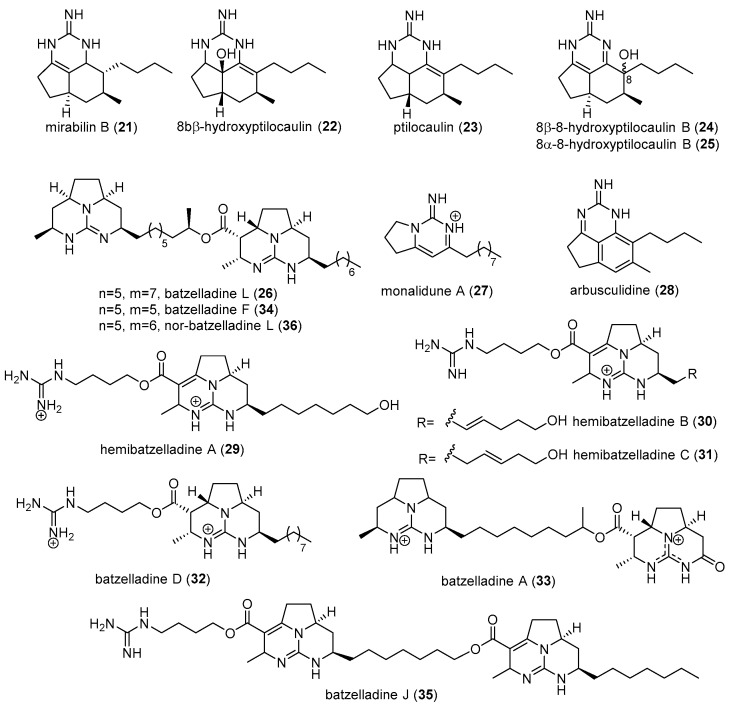
Structures of tricyclic MGAs (**21**–**36**) from sponges of the genus *Monanchora*.

The chemical diversity and complexity of the genus *Monanchora* was not only exemplified by monanchocidin-like pentacyclic MGAs, but also tricyclic ones such as batzelladines, bicyclic ones such as urpocidins, and acyclic ones such as pulchranin A. The most frequently discovered tricyclic MGAs were mirabilin analogs, which comprise a [6,6,5]-*syn*-fused tricyclic ring system with the guanidine group in the center [20]. In fact, such a tricyclic structural skeleton lacked only two spiro ether rings compared with that of pentacyclic MGAs, and thus these tricyclic MGAs were considered to be the precursor of the related pentacyclic ones (Figure 3). In 2011, by using a bioassay-guided approach, Costa-Lotufo and co-workers isolated five known tricyclic MGAs, i.e., mirabilin B (**21**), 8bβ-hydroxyptilocaulin (**22**), ptilocaulin (**23**), and a mixture of the 8β- and 8α-epimers of 8-hydroxymirabilin B (**24** and **25**), from *M. arbuscula* colonies collected off the coast of Ceará State, Brazil, at a depth of 18 m [21]. The authors focused on the biological investigation of the isolates. In the cytotoxic assay of all five compounds against four different tumor cells [HL-60, HCT-8 (human colon cancer cell), MDA-MB-435 (human breast cancer cell), SF-295 (human XG malignant glioma cell)] and one normal human peripheral blood mononuclear cell (PBMC), **22** exhibited activities against HL-60, MDA-MB-435 with IC_50_ values of 7.89 and 11.34 μM, respectively, while **23** was cytotoxic against HL-60, HCT-8, MDA-MB-435 with IC_50_ values of 5.77, 17.69, and 7.58 μM, respectively. Neither compound **22** nor compound **23** showed cytotoxicity against PBMC, which warrants further study. In fact, the authors performed a mechanistic study of the cytotoxic effect of **23** on HL-60 leukemia cells, including antiproliferative activity, morphological changes, and caspase activation using flow cytometry. **23** was confirmed to induce apoptosis of the HL-60 cells. In addition, compounds **22** and **23** were tested for hemolytic activity and showed moderate effects with EC_50_ values of 577.95 and 352.91 μM, respectively. Batzelladines are a series of tricyclic MGA dimers possessing two tricyclic ring systems connected by a long ester chain. The first examples of such a skeleton are batzelladines A-E, which were discovered by Patil and co-workers in a Bahamas sponge *Batzella sp*. in 1995 [22]. *Monanchora* sponges are rich in batzelladines. In 2014, batzelladine L (**26**) was isolated from *M. arbuscula* and was found to have antifungal activity against *Aspergillus flavus* strains with minimal inhibitory concentration (MIC) values ranging from 1.9 to 7.8 μg/mL and minimal fungicide concentrations (MFCs) ranging from 3.9 to 15.6 μg/mL [23]. These results indicated that batzelladines are potential drug leads against aflatoxigenic fungi. From the same *Monanchora* sponge species but collected off the coast of Cabo Frio, Brazil, Berlinck and co-workers isolated a total of eleven bicyclic and tricyclic MGAs after HPLC-UV-ELSD-MS-guided fractionation of its anti-parasitic extract [24]. Among them, monalidine A (**27**) is a [5,6]-fused bicyclic MGA, arbusculidine (**28**) represents the first tricyclic MGA to possess a benzene ring, and hemibatzelladines A–C (**29**–**31**) and batzelladine D (**32**) are four examples of tricyclic MGA linked to a terminal acyclic guanidine group by a long ester chain. All the others, including batzellamide A (**33**), batzelladines F (**34**), L (**26**), J (**35**), and nor-batzelladine L (**36**) are batzelladine-like tricyclic MGA dimers, of which batzelladine J (**35**) comprises two tricyclic guanidine ring systems plus one terminal guanidine group. It is worth mentioning that the authors have accomplished the total synthesis of monalidine A to confirm its structure, which will be discussed in the following section. In this research, the authors also investigated anti-leishmanial, anti-trypanosomal, and cytotoxic activities of the major compounds **26**, **27**, **32**, **34**, and **36**. All the tested compounds showed strong anti-leishmanial activities against *T. cruzi* with an IC_50_ value of 2–64 μM, and **26** was the most active compound (2 μM). With respect to the anti-trypanosomal action against *L. infantum*, batzelladine F (**34**) displayed an active IC_50_ of 4 μM, while all others performed better, with IC_50_ values of 2 μM. With respect to cytotoxicity against LLC-MK2 cells (Ganges River monkey kidney cells), only **34** exhibited moderate activity with an IC_50_ value of 10 μM, all others possessed IC_50_ values higher than 20 μM.

**Figure 4:**
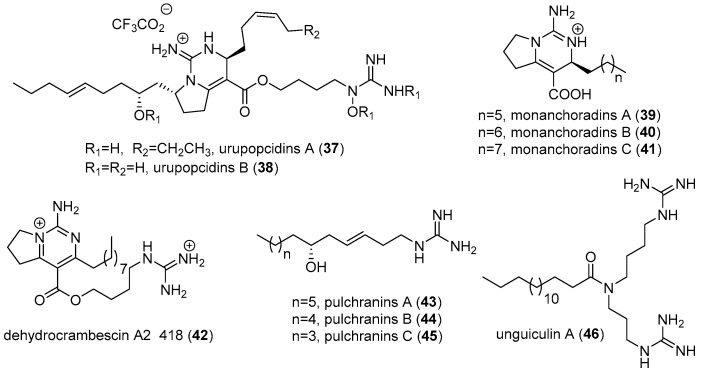
Structures of bicyclic and acyclic MGAs (**37**–**46**) from sponges of the genus *Monanchora*.

Bicyclic MGAs have been less frequently reported from *Monanchora* sponges in recent years (Figure 4). Other than the above-mentioned monalidine A, urupocidins are the represented bicyclic MGAs. They comprise a 5,6-fused cyclic guanidine ring system, which is connected to three aliphatic side chains at C-10, C-14, and C-15 positions. The C-14 side chain is linked to a terminal acyclic guanidine group. Urupocidins A and B (**37** and **38**) were first isolated by Makarieva and co-workers from the deep sea sponge *M. pulchra* near Urup Island at a depth of 183 m in 2014 [25]. Urupocidin A (**37**) was found to increase nitric oxide production in murine macrophages by inducing iNOS (inducible nitric oxide synthase) expression at 10.0 and 1.0 μM concentrations. In 2016, except for the above-mentioned pentacyclic MGAs, Ali and co-workers isolated four new bicyclic ones, monanchoradins A–C (**39**–**41**), and dehydrocrambescin A2 418 (**42**), from the French Polynesian *Monanchora* n. sp. sponge [18]. The structures of these compounds are less complex than those of urupocidins, with the similar cyclic ring system linked to only one aliphatic side chain. All four compounds were tested for cytotoxic activities against KB tumor cell lines, and **39** and **42** showed strong activities with IC_50_ values of 7.7, and 0.1 μM, respectively.

Acyclic MGAs, which have seldom been discovered in *Monanchora* sponges (Figure 4), are much less complex and diverse than the cyclic ones. The represented acyclic MGAs are pulchranins A–C (**43**–**45**), featured by a terminal guanidine with a long unsaturated aliphatic chain, which have been isolated from the far-eastern marine sponge *M. pulchra* near Urup Island at about 175 m depth by Makarieva and co-workers in 2013 [26,27]. They have been found to be the first examples of marine non-peptide inhibitors of TRPV-1 (transient receptor potential cation channel subfamily V member 1) channels with EC_50_ values of 41.2, 95 and 183 μM, respectively. In 2017, along with the above-mentioned pentacyclic MGAs, Gauvin-Bialecki and co-workers isolated an acyclic one, unguiculin A **46**, from the sponge *M. unguiculate*. It showed moderate cytotoxicity against KB cell lines and antiplasmodial activity against *P. falciparum* (3D7) with IC_50_ values of 7.66 and 12.89 μM, respectively [19].

### 2.2. Crambe

**Figure 5:**
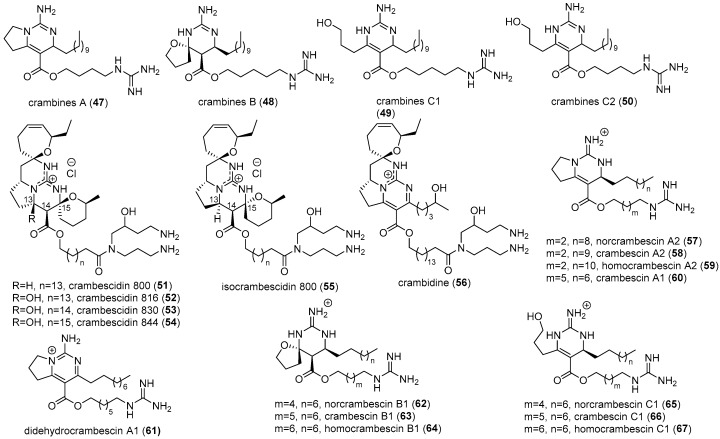
Structures of MGAs (**47**–**67**) from sponges of the genus *Crambe*.

*Crambe*, of the order Poecilosclerida and the family Crambeidae (the same as *Monanchora*), has the origin as well-known MGAs such as crambescins and crambescidins. *Crambe crambe* is a red marine sponge, mainly distributed in the western Mediterranean Sea. Braekman and co-workers completed the first chemical study of this sponge in 1990 and 1992 [28,29], leading to the discovery of crambines A (**47**, 5,6-fused bicyclic MGA), B (**48**, 5,6-spiro bicyclic MGA), C1 (**49**), and C2 (**50**). It is worth mentioning that **49** and **50** are rare monocyclic MGAs. Meanwhile, the groups of Braekman and Rinehart discovered the well-known polycyclic MGAs, crambescidins 800 (**51**), 816 (**52**), 830 (**53**), 844 (**54**), isocrambescidin 800 (**55**) and crambidine (**56**) [30,31], which attracted a lot of attention because of their total synthesis and biological studies (Figure 5). In recent years, the chemical investigation of sponges of the genus *Crambe* was not as frequent as *Monanchora,* which might be due to the previous intensive study of this genus and also because of its limited distribution. There is only one report on the chemical reinvestigation of *Crambe* by the French group of Thomas in 2012, and one biological study was conducted by the Spanish group of Botana in collaboration with Thomas in 2015. Thomas and co-workers isolated 11 crambescin derivatives (**57**–**67**), and crambescidin 816 (**52**) (Figure 5), from the Villefranche-Sur-Mer (France) at 10–20 m depth [32]. The 11 crambescins can be divided into three types. The first type, including **57**–**61**, is of a crambescin A skeleton, which is characterized by a 5,6-fused bicyclic ring system connected to one aliphatic side chain at the C-13 position and another one with a terminal guanidine group at the C-7 position. Compounds **62**–**64** represent the second type of crambescin B skeleton, featured by a [4,5]decane spiro-ring system with similar side chains as crambescin A. The last type, exemplified by **65**–**67**, is from the crambescin C family, with a monocyclic guanidine ring linked to two side chains. The absolute configurations of compounds **57**, **63** and **67** were determined by the TDDFT (time-dependent density functional theory) ECD (electronic circular dichroism) method. All the compounds were tested for their cytotoxicity against cortical neurons, with the known one **52** found to be the most toxic. In fact, crambescidins have been widely biological investigated, while there are not many biological reports on crambescins. Thus, Botana and co-workers cytotoxically examined crambescins A1 (**60**) and C1 (**66**) against human hepatoblastoma cell HepG2, including mechanistic studies, such as proliferation, apoptosis, and antioxidant activities [33]. The results revealed that **66** was able to induce MTs (metallothioneins) transcription and synthesis in HepG2 cell lines, protecting cells from oxidative damage at concentrations that retained cell viability. Such an observation provided the first comprehensive method regarding the various bioactivities of crambescins on tumor cells and gave a biological clue for the future investigation of such compounds.

### 2.3. Other Sponges

**Figure 6:**
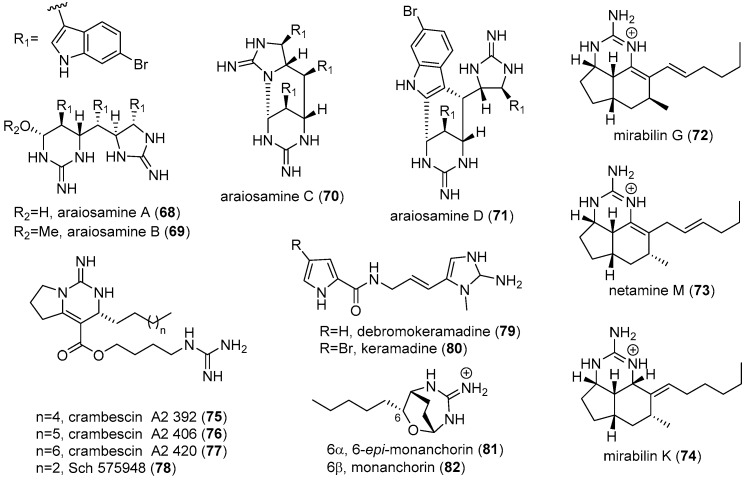
Structures of MGAs (**68**–**82**) from the other marine sponges.

Although numerous complex MGAs were obtained from *Monanchora* and *Crambe*, their diversity is not that wide. Marine chemists turned to the chemical investigation of other sponges from different locations such as those from the genus *Clathria* in Vanuatu, *Halichondria* in Okinawa, *Pseudaxinella* in Bahamas, *Acanthella* in Australia, *Agelas* in Solomon Islands, etc. In 2011, Wagoner and co-workers isolated four new tris-bromoindole cyclic guanidine alkaloids, araiosamines A–D (**68**–**71**) (Figure 6), from the marine sponge, *Clathria* (Thalysias) *araiosa*, collected from Vanuatu [34]. The structures of these compounds are totally different from the aforementioned MGAs. They comprise three bromoindole rings, connecting to six-membered and five-membered guanidine rings. The authors tested the phenotypic effects against zebrafish embryos, antibacterial activity against *Staphylococcus aureus*, and HIV infection of the araiosamines, without showing any significant activities. Nevertheless, araiosamines represent a novel scaffold of MGAs discovered from marine sponges. Typical tricyclic MGAs, mirabilin G (**72**), netamine M (**73**), and mirabilin K (**74**), have been obtained from the Southeastern Australian sponge Acanthella Cavernosa [35]. These mirabilin derivatives were evaluated for activity against tumor suppressor PDCD4 (Programmed cell death protein 4), of which **72** and **73** displayed strong inhibitory activities in the cellular degradation of PDCD4 with EC_50_ values of 1.8 and 2.8 μg/mL, respectively. To the authors’ best knowledge, **72** and **73** are the first MNPs reported to stabilize PDCD4 under tumor promoting conditions. Bicyclic MGAs of the crambescin skeleton, exemplified by crambescins A2 392 (**75**), 406 (**76**), 420 (**77**), and Sch 575948 (**78**), were discovered in the Bahamas sponge *Pseudaxinella reticulate* collected in Sweetings Cay at a 17-m depth (Figure 6) [36] by Molinski and co-workers in 2015. All the compounds were tested for antifungal activity against the pathogenic strains *Candida albicans* and *Cryptococcus* sp., with MIC_50_ ranging from 1.1 to 39 μg/mL and MIC_90_ from 2.3 to 59 μg/mL. The length of the alkyl side chain was responsible for the variation of activities. In the same year, the group of Ali and co-workers investigated a Pacific sponge *Agelas cf. mauritiana* from Solomon Islands at 18–30 m depth, resulting in the isolation and elucidation of debromokeramadine (**79**) (Figure 6) [37]. Compound **79**, belonging to the pyrrole-2-aminoimidazole (P-2-AI), is a debromo product of keramadine (**80**), which was isolated by Kobayashi and co-workers from an Okinawan Sea sponge *Agelas* sp. [38]. The authors achieved total synthesis of **79** and **80** through a key regioselective oxidative addition of a guanidine derivative on *N*-acylpyrrole dihydropyridines. This method was applied for the synthesis of the analogs of **79**, which allowed the further biological study of P-2-AI derivatives. In 2016, two 6,7-fused bicyclic MGAs, 6-epi-monanchorin (**81**) and its known isomer monanchorin (**82**) (Figure 6), were isolated by Yamazaki and co-workers from the Okinawan marine sponge *Halichondria panicea* at Iriomote Island in Japan [39]. Neither compound exhibited activity against *M. segmatis* at 20 μg/disc nor cytotoxicity against cancer cell lines at 10 μM.

## 3. MGAs from Other Marine Sources

**Figure 7:**
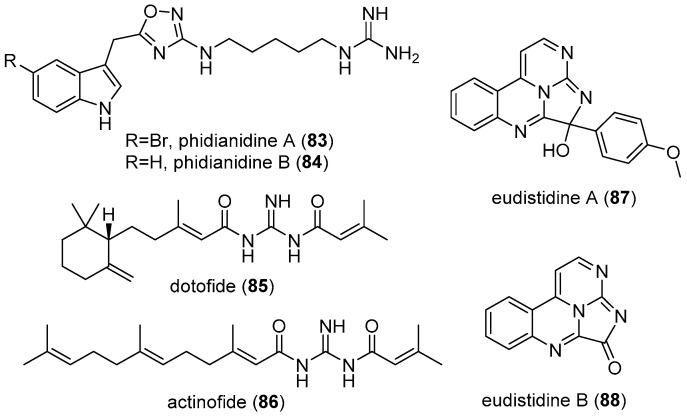
Structures of MGAs (**83**–**88**) from other marine sources (mollusks and ascidian).

Despite that most of MGAs are of sponge origins, a few of them were found in other marine resources. Apparently, during the last seven years, there is no report on guanidine-containing toxins related to TTX or STX from marine algae, while a few miscellaneous MGAs were obtained from marine mollusks and ascidians (Figure 7). In 2011, in collaboration with the group of Gavagnin in Italy, our group isolated two novel guanidine-containing indole alkaloids, phidianidines A and B (**83** and **84**), from the South China Sea opisthobranch mollusk *Phidiana militaris* in Hainan Island, China. Phidianidines comprise an unprecedented 1,2,4-oxadiazole connected to an indole ring and a long side chain bearing a terminal guanidine group [40]. They showed high cytotoxicity against tumor and nontumor mammalian cells with IC_50_ values ranging from 0.14 to 100.2 μM. Due to the novel structures and the high biological activities, phidianidines attracted a lot of attention for their synthesis, which will be discussed in the next section. Biologically, it is worth mentioning that phidianidine A (**83**) was identified as a new CXCR4 (C-X-C chemokine receptor type 4) ligand for antagonist activity by Amodeo and co-workers in 2013, with various intensive biological studies to confirm their results [41]. In the same year, König and co-workers isolated a novel guanidine-interrupted terpenoid, dotofide (**85**), from the marine mollusk (slug) *Doto pinnatifida* (Gastropoda, Nudibranchia) collected in Ferrol, Spain. The authors speculated that such a terpenoid-MGA hybrid could probably act as a chemical defensive metabolite for shell-less mollusks like *D. pinnatifida* [42]. Recently, once again, our group and the group of Gavagnin collaborated with each other and discovered a new diacylguanidine, actinofide (**86**), from the South China Sea mollusk *Actinocyclus papillatus* collected in Wei Zhou Island, China [43]. Actinofide is a terpenoid-guanidine hybrid, featured by a guanidine group acylated by two terpenoid acid units. Furthermore, a four-step synthesis of actinofide and its analogs was achieved, which will be discussed in the next section.

Marine ascidians are also a very important source of bioactive marine alkaloids. One of the most famous marine natural product-derived drugs, ecteinascidin-743 (ET-743), under the trade name Yodelis by PharmaMar/Johnson & Johnson/OrthoBiotech for the treatment of refractory soft-tissue sarcomas, which was approved by the European Commission in 2007, was first isolated from the marine tunicate *Ecteinascidia turbinate* [44,45]. In recent years, only one report has been observed for the MGAs from ascidians. By chemical investigation of the colonial marine ascidian *Eudistoma* sp. collected in Koror/Airai Channel, Palau, Gustafson and co-workers isolated two novel polycyclic MGAs, eudistidines A and B (**87** and **88**), using a bioassay-guided approach [46]. **87** and **88** possess an unprecedented tetracyclic core comprising a fused pyrimidine, quinazoline, and imidazole ring, in which a guanidine functionality is embedded. In a bio-screening assay, **87** effectively blocked the binding of soluble CH1 (the protein binding domain of transcriptional coactivator protein p300) to immobilized C-TAD (the protein binding domain of transcription factor HIF-1: hypoxia-inducible factor 1), with IC_50_ of 75 μM, while **88** was not active. The structure of **87** may provide a new chemical scaffold to study p300/HIF-1α interactions towards novel anticancer drug discovery.

**Figure 8:**
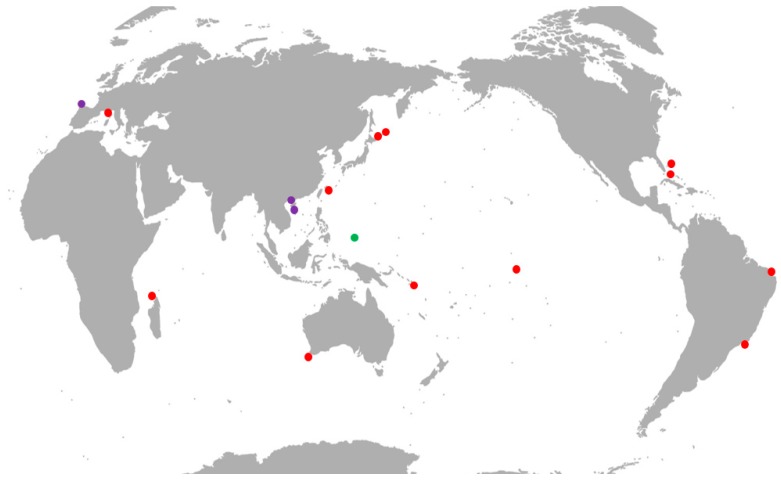
The global distribution of the MGA-containing marine organisms, which have been chemically investigated since 2010 (red means sponges, violet means mollusks, green means ascidians).

**Figure 9:**
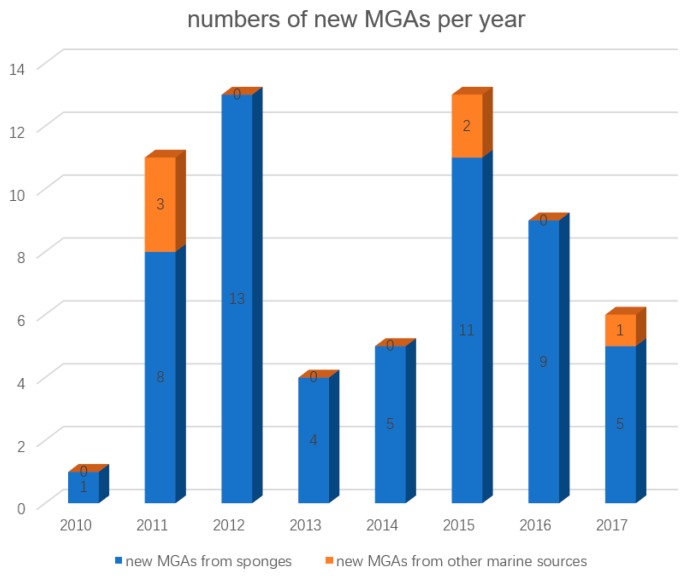
New MGAs from 2010 to 2017 (2017 data were updated on 30 June 2017).

In summary, although guanidine containing metabolites are extremely rare in nature, more than 60 MGAs have being isolated since 2010 from various marine sources located all over the global ocean (Figure 8), with sponges as the most frequent origins. As shown in Figure 9, only 3, 2 and 1 MGAs were isolated from other marine sources, including mollusks and ascidians, in 2011, 2015, and 2017, respectively. These data indicated that marine sponges are the main sources of MGAs in recent years, which should be attributed to their relatively larger amount and ease of collection.

## 4. Synthetic Examples of Recently Discovered Bioactive MGAs

The beautiful structures and broad biological activities of MGAs have unquestionably attracted many synthetic chemists with respect to their total syntheses. A range of well-known and complex MGAs have been synthesized many times using different approaches, such as the toxin family TTX and STX, pentacyclic crambescidins, tricyclic batzelladines, bicyclic urpocidins, etc. [45,46,47,48,49]. In recent years, some of them, even those discovered in nature more than 10 years ago, still underwent synthetic studies, such as the polycyclic dragmacidin E, massadine, axinellamines, and the TTX derivatives [50,51,52,53]. In light of the review length, we will only discuss the synthesis of the abovementioned new MGAs which have been isolated since 2010.

### 4.1. Monalidine A

**Scheme 1:**
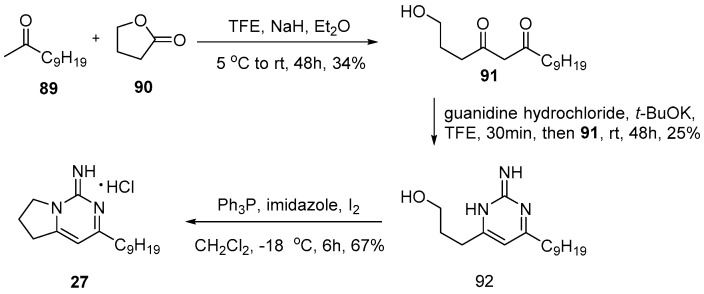
Total synthesis of monalidine A (**27**).

After the isolation and structure determination of monalidine A (**27**), Berlinck and co-workers decided to synthesize this natural MGA to confirm its structure and to accumulate an amount for biological study. Starting with undecan-2-one (**89**), Claisen condensation with dihydrofuran-2(3*H*)-one (**90**) in the presence of sodium hydride (NaH) and trifluoroethanol (TFE) in Et_2_O was performed, producing 1-hydroxypentadecane-4,6-dione (**91**) at a yield of 34% (Scheme 1). The generated guanidine free base from guanidine hydrochloride in the presence of *t*-BuOK in anhydrous TFE was then reacted with **91** to produce pyrimidine (**92**) in 25% yield. It was finally cyclized in the presence of a modified Mitsunobu protocol to produce monalidine A (**27**) as its hydrochloride salt with 67% yield. Thus, a concise three-step synthesis of **27** was achieved with 5.7% overall yield by using inexpensive starting materials [24].

### 4.2. Araiosamines

**Scheme 2:**
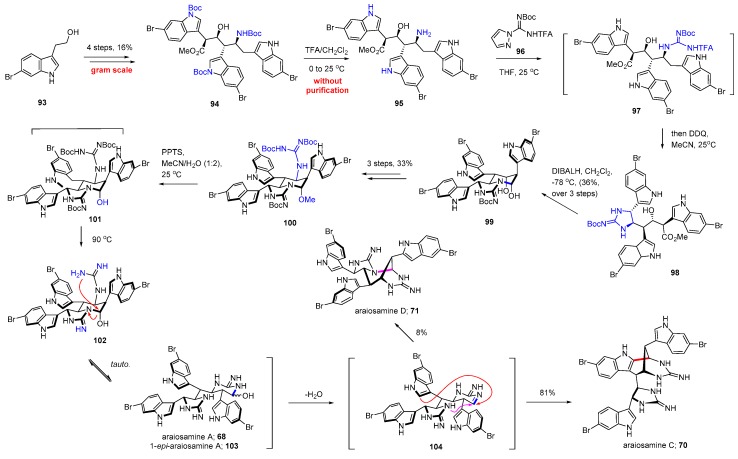
Baran’s total synthesis of araiosamines A (**68**), C (**70**) and D (**71**).

Araiosamines were discovered by Wagoner and co-workers with remarkable structures, including six continuous stereocenters, two guanidine units, three bromoindole heterocycles and, in araiosamines C and D (**70** and **71**), unique bicyclo [3.1.1] ring systems [34]. These molecules attracted the attention of Baran’s group for their synthetic studies. An 11-step concise total synthesis of araiosamines A, C and D (**68**, **70** and **71**) was finally accomplished by Baran in 2016 (Scheme 2), with key steps involving an impressive selective C–H functionalization, a new reagent for guanidine installation, and an amazingly simple final step that intersects a plausible biosynthetic intermediate [54].

In detail, starting with 6-bromo-tryptophol (**93**), a first 4-step reaction performed well to produce **94** with approximate 16% overall yield. After obtaining the free amine **95** by treating **94** with TFA, a challenge pertaining to the installation of the first guanidine occurred. Several guanidinylation reagents were attempted and gave negative results, and finally *N*-Boc,*N*′-trifluoroacetyl-guanylpyrazole (**96**) was synthesized and enabled the reaction with **95** towards the intermediate **97**. It was then treated by 2,3-dicyano-5,6-dichlorobenzoquinone (DDQ) to promote a “Yonemitsu-type” oxidation of the C-6 indole for a pivotal chemo- and stereoselective C-H functionalized cyclization to afford **98**, which underwent DIBAL reduction to produce **99** with 36% yield over the three steps. **99** underwent oxidation, reductive amination, reduction, and second guanidinylation to obtain the key intermediate **100** with 33% global yield. Hydrolysis of **100** with pyridinium *p*-toluenesulfonate (PPTS) in MeCN/H_2_O at room temperature over 3 days yielded **101**, which was heated over 90 °C to remove the Boc protecting groups to liberate **102**. Intriguingly, **102** was found to exist in equilibrium with araiosamine A (**68**) and 1-*epi*-araiosamine A (**103**) via ring chain tautomerization, and the isolated **68** could revert back to a tautomeric mixture of **102** and **103** over several hours. According to the supporting information of Baran’s *JACS* paper [54], a continued heating of this mixture for 24 h yielded araiosamines C (**70**) and D (**71**) through the intermediate “pre-araiosamine” (**104**). Therefore, by using fantastic chemical reactions, Baran and co-workers achieved the total synthesis of araiosamines A, C and D in 11 formal steps with the overall yield of 1.94%, 1.57%, and 0.16%, respectively.

The synthetic ±araiosamines A, C and D were also tested for cytotoxic activities, and similar to the natural ones, no activity was found against human carcinoma cell lines BT-474 and HCC1954. However, despite that the natural araiosamines were reported to be inactive against the Gram-positive bacteria *Staphylococcus aureus*, the synthetic ones displayed considerable activities against both *S. aureus* with MIC of 1–2 μg/mL, and the Gram-negative *E. Coli* with MIC of 2–16 μg/mL. We presume that this difference might be attributed to the racemic properties of the synthetic ones, and thus the optical pure compounds should better be used for future biological evaluation.

### 4.3. Keramadine and Debromokeramadine

As members of the P-2-AI family, keramadine and debromokeramadine were synthesized by Ali and co-workers immediately after their discovery [37]. Although there are already a few reports on the synthesis of keramadine, N1-methyl 2-AI construction was always a main challenge due to the low selectivity and yield of the natural products.

**Scheme 3:**
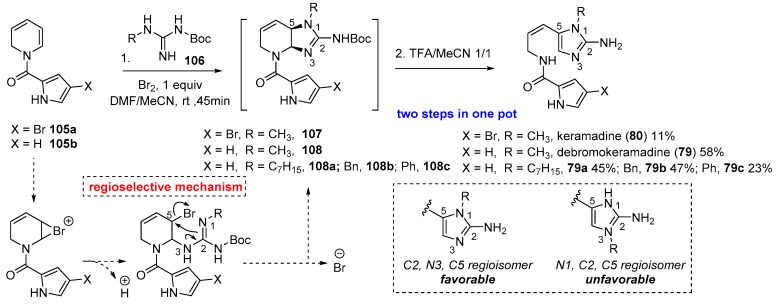
The synthesis of keramadine, debromokeramadine, and their analogs by a one-pot two-step reaction with regioselectivity.

Interestingly, the authors created a flexible one-pot synthesis towards the two natural products (Scheme 3). They started from the previously described *N*-acylpyrrole-1,2 dihydropyridine **105**, followed by a regioselective reaction, favorable for C2, N3, C5 regioisomer, with *N*-methyl-*N*′-Boc-guanidine (**106**) in the presence of 1 equiv bromine, giving the expected bicyclic products **107**, **108**, and **108a**–**c**. An attempt to treat these regioselective intermediates under acid conditions led to the direct cleavage of the amino bond and the Boc deprotection towards the aim of natural products **79** and **80** in one-pot synthesis. By applying the same approach, the authors were able to synthesize a series of **79** analogs (**79a**–**c**). The authors intended to prepare more analogs for further biological evaluation and structure-activity relationship (SAR) analysis.

### 4.4. Actinofide

**Scheme 4:**
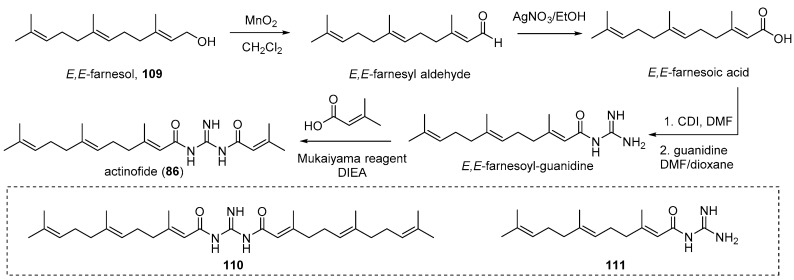
The synthesis of actinofide and analogs.

Immediately after the discovery of actinofide from the mollusk *Actinocyclus papillatus* [43], our group and Gavagnin’s group completed a four-step synthesis of this natural product (Scheme 4) and its analogs for further biological investigation. The total synthesis started with *E,E*-farnesol (**109**), which went through two-step oxidation, guanidinylation, and amidation to yield actinofide (**86**) and analogs in high quantities [43].

All the synthetic compounds, along with an isolated guanidine analog dotofide (**85**) from *D. pinnatifida* in Naples, were subjected to in vitro growth inhibitory activity against six cancer cell lines with distinct historical origins, including two human carcinoma cancer cell lines of epithelial origin [MCF7 (breast cancer) and A549 (nonsmall-cell lung cancer; NSCLC)], two human cancer cell lines of glial origin [Hs683 oligodendroglioma, and the U373 glioblastoma of astrocytic origin models], and two melanoma models (the mouse B16F10, and the human SKMEL-28). Among them, **86** and **110** showed the most antiproliferative activities against cancer cells of glioma origin (Hs683 and U373), with IC_50_ of 6.8–15.7 μM; **85**, **86**, **110**, and **111** performed better than the others against cancer cells of carcinoma origin (A549 and MCF7) with IC_50_ around 20 μM; and the above four compounds also exhibited stronger activities against B16F10 cells of melanoma origin with an IC_50_ value less than 10 μM. The results revealed that the size of the terpenoid units influenced the degree of activities, and the cyclic farnesoyl moiety of **85** had a negative influence on the activity, compared with its corresponding acyclic actinofide **86**. Although the synthetic approach is simple, it is significant to rapidly obtain such MGAs for further extensive biological evaluation and SAR study, and it should be noted that dimethyldiguanide is a well-known case of a simple compound-derived drug.

### 4.5. Phidianidines

**Scheme 5:**
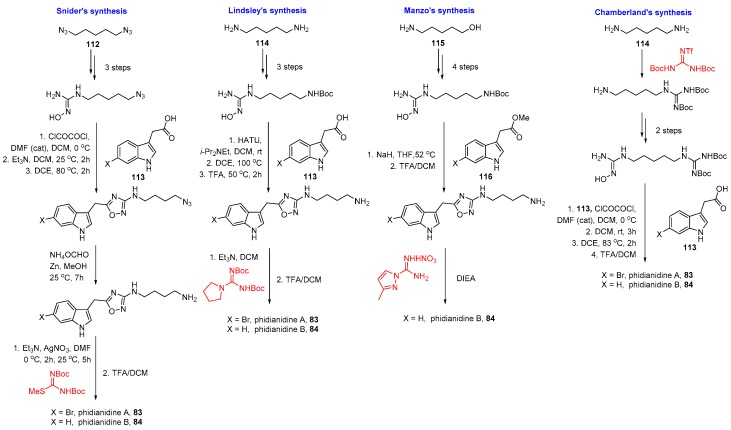
The four different routes for the syntheses of phidianidines.

Phidianidines are a series of remarkable MGAs with an unprecedented 1,2,4-oxadiazole ring, and we would like to highlight their syntheses in this review. Phidianidines were also discovered by our group and Gavagnin’s group in 2011 from the mollusk *Phidiana militaris* [40]. Since then, many groups endeavored to synthesize these structurally intriguing and biological active MGAs. One year after the discovery of phidianidines, three groups accomplished their total synthesis independently (Scheme 5), including Snider’s group [55], Lindsley’s group [56], and Manzo’s group [57].

Snider and co-workers started with diazide **112** and indole-3-acetic acid (**113**), carried out via a key reaction for the construction of the 1,2,4-oxadiazole ring followed by late-stage guanidinylation, and finally installed the natural products in eight steps with 19% overall yield [53]. This paper was the first report of the total synthesis of phidianidines. Recently, Lindsley and co-workers reported the synthsis of phidianidines and their analogs, as well as their biological study, in *ACS Chem. Neurosci.* [56]. The synthetic method is similar as that of Snider, while 1,5-diaminopentane **114** was used as the starting material, the guanidinylation was also proceeded at a late state after the cyclization of the oxadiazole ring. This synthesis only consumed six steps with 39.9% and 21% overall yield for **83** and **84**, respectively. Via the biological evaluation, **83** and **84** were found to be potent ligands for the μ-opioid receptor with high selectivity, since no activity against the δ- or κ-opioid receptors were observed, and both compounds exhibited weak partial agonist μ-opioid activity. They have also been found by the authors to be selective inhibitors of DAT (dopamine transporter), versus SERT (serotonin transporter) and NET (norepinephrine transporter). Interestingly, **83** and **84** were not cytotoxic against Human Embryonic Kidney 293 (HEK293) cells. Therefore, **83** and **84** could be further studied towards potent and selective ligands for CNS (central nervous system) targets of therapeutic relevance. The authors also synthesized some structural analogs of phidianidines, but no biological data are available for them. Soon thereafter, Manzo and co-workers started with 5-amino-1-pentanol (**115**) and methyl indole-3-acetate (**116**) and synthesized phidianidine B (**84**) in seven steps with 14% total yield [57]. Different from the above two methods, the late-stage guanidinylation of Manzo was achieved by using 3,5-dimethyl-1-pyrazolylformaminidium nitrate. In 2013, Chamberland and co-workers reported a concise synthesis of phidianidines A (**83**) and B (**84**) [58], featured by the construction of the guanidine group at the early-stage, before the formation of the oxadiazole ring. The total synthesis, starting from 1,5-diaminopentane (**114**), was accomplished, with 5.4% and 5.7% for **83** and **84**, respectively.

**Figure 10:**
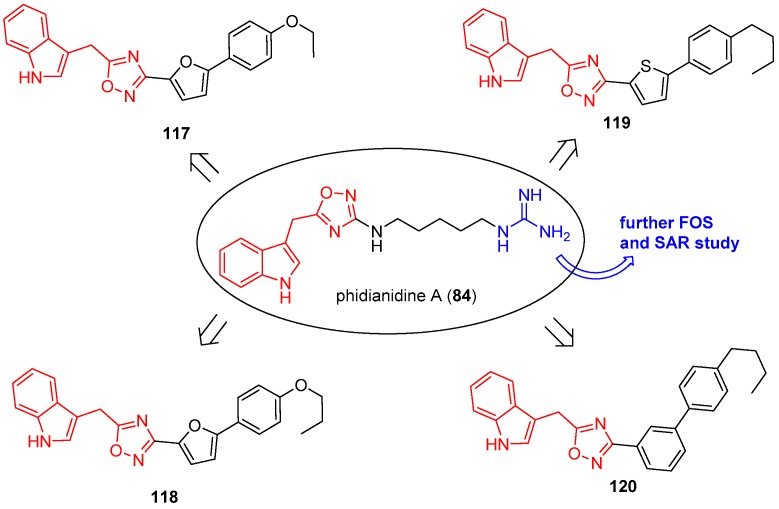
Representative bioactive compounds synthesized by our group.

In light of various total syntheses and biological studies, the phidianidine scaffold became popular in both synthetical and biological communities. Our group has long been engaged in the design and function oriented synthesis (FOS) of phidianidine analogs towards more biological active compounds. The initial strategy was to keep the indole and oxadiazole skeleton but remove the long guanidine chain. Two series of such derivatives with various heterocycles to replace the guanidine chain were synthesized by using metal catalyzed couplings. A series of the analogs, exemplified by **117**, and **118**, showed considerable neuroprotective effects against amyloid-β_25–35_ (A β_25–35_)-, hydrogenperoxide (H_2_O_2_)-, and oxygen-glucose deprivation (OGD)-induced neurotoxicity in SH-SY5Y cells, with cell viability of 80–120%, comparable with the positive control [59]. Another series of derivatives, such as **119** and **120** (IC_50_ = 8.96 and 8.80 μM, respectively), exhibited significant PTP1B (ProteinTyrosine Phosphatase-1B) inhibitory activities towards anti-diabetes drug design [60]. The systematic FOS of phidianidine analogs by our group to further evaluate the function of the guanidine group is still ongoing on the basis of our previous results and with the aid of computer-assisted drug design (Figure 10).

## 5. Conclusions and Perspectives

In this review, we summarized the isolation, synthesis and biological activities of diverse and complex marine guanidine-containing natural products from the year 2010 to the present (Figure 9). Most of these remarkable MGAs were isolated from marine sponges all over the world (Figure 8), especially the genus *Monanchora*, with structures ranging from the simplest acyclic core to the most complex pentacyclic skeleton. It is obvious that the MGAs have been much less discovered from marine mollusks than sponges, at least since 2010, which might be due to the difficulty of the sample collection (Figure 8). With respect to soft corals, although some interesting sulfur-containing alkaloids have been isolated in recent years [61], no MGAs were found in this kind of animal. With the chemo-ecological research experience related to marine mollusks over the years, we suspected that the guanidine derivatives from these animals could possibly originate from their sponge preys. In particular, the mollusks ate their dietary sponges, accumulated the guanidine secondary metabolites from the sponges, digested those complex MGAs to be small building blocks, and finally transferred them into their own metabolites by self-assembly, such as the above-mentioned terpenoid-MGA hybrids dotofide (**85**) and actinofide (**86**). That would probably be the reason why the MGAs from mollusks have become much simpler than those from sponges. It would be intriguing and significant to find the prey of those guanidine-containing mollusks and to study their chemo-ecological relationship for a reference of further chemical and biological investigation of those bioactive MGAs.

In view of the isolation of numerous MGAs year after year with various biological activities, some of them would have the potential to be drug candidates in clinical trials in the near future [62]. Given the poor understanding of the mechanisms of bioactive MGAs at the molecular level, the application of advanced chemical biological methods, combined with rapid synthetic skills, would speed up the development of MGAs towards novel drug leads.
